# Resource acquisition in diel cycles and the cost of growing quickly

**DOI:** 10.1371/journal.pcbi.1013132

**Published:** 2025-06-06

**Authors:** Kevin J. Flynn, Andrew Yu. Morozov

**Affiliations:** 1 Plymouth Marine Laboratory, Plymouth, United Kingdom; 2 School of Computing and Mathematical Sciences, University of Leicester, Leicester, United Kingdom; 3 Institute of Ecology and Evolution, Russian Academy of Sciences, Moscow, Russia; University of Toronto, CANADA

## Abstract

Many organisms, notably phototrophs, routinely acquire resources over only a fraction of the day. They have to balance their main period of initial biosynthesis against cell cycle events. Because of their short generation times, this challenge is especially acute for the planktonic microalgae that perform 50% of global C-fixation. Empirical evidence indicates that microalgal day-average growth is a function of the ability to acquire resources rapidly when available, retaining initial products of assimilation to support growth. A fundamental question arises over the optimal physiological configuration to support such activity. Here, we applied computer simulations implementing a development of the quota concept, in which the internal limiting resource is itself C, ratioed against total organism C-biomass. The model comprises metabolite and core pools of carbon C (^*M*^*C* and ^*C*^*C*, respectively), with growth modulated by ^*M*^*C*/(^*M*^*C*^* *^+ ^*C*^*C*); ^*M*^*C* supports growth of ^*C*^*C* in the absence of concurrent resource acquisition. Dynamic feedback interactions from the relative size of ^M^C controls resource acquisition. The model reproduces the general pattern of growth at different light:day fraction (*LD*), and of afternoon-depression of C-fixation. We explored the efficiency of the physiological cell configuration to locate optimal configurations at different combinations of maximum growth rates (*U*_*max*_) and *LD* values across plausible parameter values for microalgae. While the optimum maximum resource acquisition rate deployed during the L phase scales with *U*_*max*_/*LD*, the maximum size of the metabolite pool scales to *LD*/*DV*, where *DV* is division time (i.e. *U*_*max*_/Ln(2)). Accordingly, we conclude that faster growing organisms carry a penalty limiting their geographic spread to latitudes and seasons where *LD* is high. Larger, vacuolated organisms (such as diatoms), having a bigger metabolite compartment, may be at an advantage in such situations.

## Author summary

Planktonic microalgae that support 50% of global primary productivity have a problem: for half of their growing lives, during night time, they cannot fix C. We explored how they may optimise C-fixation during the illuminated fraction of the day (*LD*) to support biosynthesis over the whole day. We constructed a model describing a metabolite pool, into which the initial results from C-fixation accumulate, and a core structural pool. Consistent with how real organisms function, the relative size of the metabolite pool modulates, or controls, not only the synthesis (growth) of the core structure, but also modulates C-fixation; both modulations used sigmoidal functions in line with allosteric biochemical controls. We found that, while the C-fixation rate required to support a given growth rate potential (*U*_*max*_) increases broadly linearly with 1/*LD*, the relationship with the maximum size of the metabolite pool relative to the whole organism biomass (*M*_*max*_) relates to the ratio of *LD* to the organism’s doubling time. Faster growing organisms thus need not only a higher resource acquisition rate (C-fixation) for a given combination of *LD* and *U*_*max*_, but also a larger *M*_*max*_. We suggest this limits the competitiveness of faster growing organisms to lower latitudes and/or longer day-light periods.

## Introduction

The growth of organisms may be expected to be most efficient when it proceeds under steady-state environmental conditions, because such conditions enable the organism to balance out all biochemical functionality to minimise stress and maximise the allocation of resources to growth. It is for this reason that steady-state conditions are exploited in biotechnological applications [1]. In nature, however, organisms invariably grow in non-steady-state environments, not least because of the impacts of the light-dark cycle. This is especially problematic for organisms which have generation times of around 1d, such as microalgae. Indeed, the light-dark cycle often entrains cell division [2,3] such that microalgae with a doubling time of around 1 d (i.e., with a specific growth rate, *U*≤ Ln(2)) partition resource acquisition and the cell division cycle stages between the light-dark periods. However, various microalgae can grow faster than this and the interactions between resource acquisition and growth will be more complex. Although one may suspect that the fastest growth rates would only be attainable in continuous light, some species can achieve extremely high growth rates in light-dark cycles (e.g., [4]).

Short-term acquisition of resources at rates far in excess of those required to match their average needs are common. For example, the uptake of inorganic nutrients into microbial plankton can exceed their day-average needs by many times [5,6]. However, most simulation models of these organisms assume an equilibrium interaction between resource acquisition and growth, such that *de facto* the maximum rate of acquisition defines or aligns with the maximum growth rate (e.g., [7]). This convention is born from empirical studies in which the light:day cycle ratio is held constant, where this diel equilibrium condition is inevitably met. The mismatch between simple model assumptions and reality gives rise to various challenges when considering the dynamics of growth in non-steady-state natural systems [8]. In contrast to the description in models, real organisms have to modulate resource acquisition to balance their needs. How they do so, and the fidelity with which they do so, may be expected to differ with the type of resource and the variability in availability. Collectively these traits will also help to define optimisation for organisms that evolve in different conditions; the representation of these traits are thus important topics for consideration in models.

In computational modelling of microbial growth, simulating the disconnect between resource availability and growth is well known to be important, with the simple classical model of Monod [9] being less able to describe growth dynamics than quota models [10,11]. Quota models relate growth to the internal resource availability, describing that availability either as a cell-quota or C-quota (e.g., for N-limited growth, as N cell^-1^ or N C^-1^). Nutrients that contribute only a minor part to biomass will occupy little volume in an organism, and may be accumulated to a large excess (e.g., P accumulating as polyphosphate, see [12]). The importance of modelling transient uptakes [13] has been recognised for many decades [14]. Such accumulations can support C-specific growth to continue for even several generations in the absence of concurrent resource acquisition. At the other end of the spectrum, obtaining the most important component, namely carbon, presents a challenge to phototrophs because irradiance and thence the ability to acquire the resource varies over the day light-dark cycle, seasonally with the time of year and also with latitude. Near the equator the light:day (*LD*) period is close to 0.5, while at high latitudes it varies between 0 (total darkness in winter) and 1 (continuous light in summer). Models that describe the accumulation of storage C include those of [15] and [16], though this feature is also described by the excess of C over the minimum C:N quota using a standard quota model [17]. The model of Zonneveld and collaborators [18], however, proposed a C-quota model that considered two C pools, transient and structural pools, with their concentrations expressed against the cell volume, while the model concept presented in [19] describes models using metabolic and structural pools with their concentrations expressed against the total organism-C.

**Fig 1:**
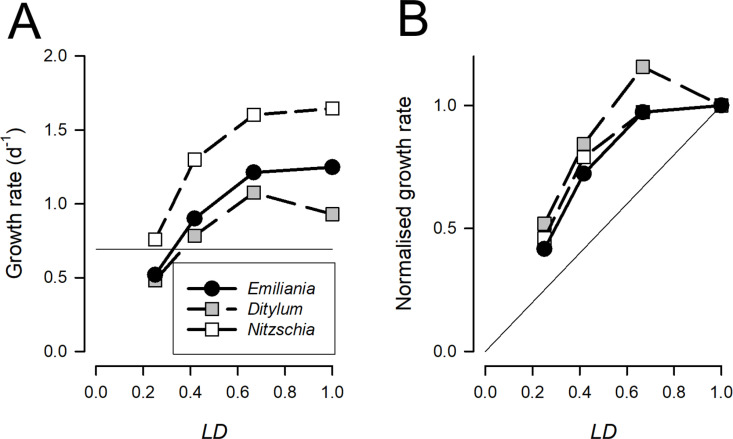
Panel (A) shows experimental data from [20,21], showing growth rates of the microalga *Emiliania* (formally *Coccolithus*) *huxleyi* and the diatoms *Ditylum brightwellii* and *Nitzschia turgidula* under saturating photon flux density (PFD) but delivered with different diurnal light periodicities (*LD*, where 1 is continuous illumination). In panel (**B**) these growth rates are shown normalised to the rate at *LD* = 1. The thin line in panel (**A**) indicates a day-average growth rate of 0.693 d^-1^ (i.e., Ln(2)) equating to a cell division per day. The thin line in panel (**B**) indicates the growth rate expected if it increased linearly with *LD*.

A crude expectation for the growth of phototrophs, such as the unicellular planktonic microalgae that support food-webs throughout the sunlit waters of the oceans, is that they grow at a maximum rate during the daylight hours and *de facto* shut down in darkness. Accordingly, we may expect to see a simple relationship between the day-average growth rate (e.g., C C^-1^ d^-1^) and *LD*. However, empirical evidence does not support such a simple relationship. For example, the data of Paasche ([20,21]; Fig 1A) reveals an ability to totally compensate for *LD* values down to ca. 0.6. Thus, microalgae grow at a day-average rate much faster with an irradiance cycle of 0.5, versus that at *LD* = 1, than the 50% day-exposure to light may be expected to support (Fig 1B). Such abilities are likely to be important factors affecting species succession [22].

The explanation to this paradox must, at its heart, require a rate acquisition (here, as C-fixation as C C^-1^ hr^-1^) during the day-light hours that exceeds the day-average requirement. However, while a model can be operated that simply describes such a high rate of resource acquisition, this raises the question as to why the organism itself should not exploit that high rate to achieve an even faster day-average rate of acquisition at *LD* = 1. The conclusion is that there must be a pinch point limiting the biochemical processing of the resources, associated at least in part with DNA-replication cell-cycle events. The partially assimilated resources that are accumulated during the light-phase must thus be held in a pool, noting that this ‘pool’ is not likely to be a single spatially resolvable entity within the organism, but rather a description of part assimilated metabolites and sub-structures. The content of this pool then supports growth of what we may term the core biomass over the whole day. Under conditions where the periodicity of resource availability (here, light within *LD* for enabling photosynthesis) may be restrictive, this intermediate pool enables a surplus to be acquired which supports growth of core physiological processes over the whole day. The dynamics of filling and exploiting (draining) that intermediate pool must also potentially be restrictive if acquisition exceeds the needs for the day-average maximum growth rate.

Here we consider the simplest situation where resource availability when it is available (e.g., light during day-light hours) is not limiting, nor (e.g., for high light) is it inhibitory. We thus have 4 interacting factors to consider that have overarching control on the dynamics: the maximum growth rate (denoted here by *U*_*max*_), the relative rate of resource acquisition rate required to satisfy *U*_*max*_ (denoted by *A*_*0*_), the size of the intermediate (metabolite) pool relative to the total organism biomass (denoted by *R*), and the value of *LD*.

Previous studies have considered the effects of resource-limitation under continuous light or a fixed *LD* ([8,16]), as did the work [18] using a multiple C-pools model of microalgae. Our work thus appears to be the first to have considered C-acquisition under variable diel cycles. Another novelty in this regard is that we exploit feedback processes to mimic the homeostatic regulations that modulate resource acquisition and growth in real organisms. The aim of this work is to explore the inter-relationships between these factors, to locate the optimal configurations required to handle different *LD*-supply patterns of resource acquisition, and to establish how the maximum growth rate of the organism may affect these configurations. These matters are important because allocating unwarranted resources to aspects of organism physiology that bring in and then at least partially assimilate resources, and also the maximum size of the metabolite pool, could counter the advantage of deploying the mechanism. These results would be crucial for understanding patterns of the geographic spread of phototrophs in various latitudes across seasons, and may affect the competitive advantage of organisms to grow under climate change scenarios that see pole-ward shifts in distribution [23].

## Materials and methods

**Fig 2:**
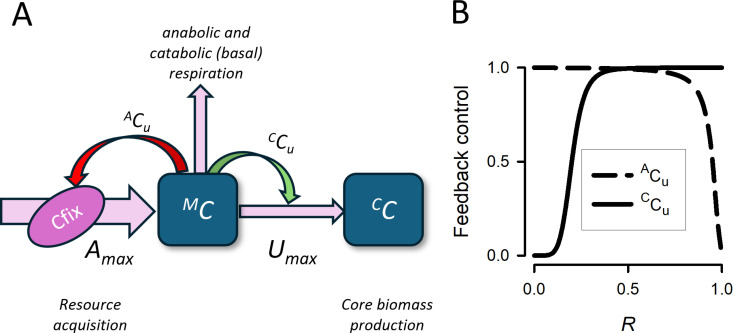
(A) The flowchart of the model. The C-biomass comprises a metabolic pool (^*M*^*C*) and a structural core (^*C*^*C*). Resource, here as C-fixation, enters at a maximum rate set by *A*_*max*_ (per capita) and is constrained by feedback from the size of ^*M*^*C*, as controlled by a sigmoidal term, ^*A*^*C*_*u*_. Resources from ^*M*^*C* are used to make ^*C*^*C* at a maximum rate set by the maximum organism growth rate, *U*_*ma*x_, constrained by the size of ^*M*^*C* via sigmoidal term ^*C*^*C*_*u*_. The resource acquisition rate is potentially higher than the core biomass production rate, hence the difference in the horizontal arrow thickness. Respiration costs are withdrawn to support catabolic (basal) and anabolic activities (see the main text for details). **(B)** Typical behaviour of the feedback functions ^*A*^*C*_*u*_ and ^*C*^*C*_*u*_ (defined by (Eqs 5) and (6)) plotted against the relative size of the metabolite pool *R* defined by (Eq 4). Full description of model parameters and model functions used is given in Table 1.

The flowchart of the model is shown in Fig 2A. The model variables, functions and parameter values used in the model are summarised in Table 1. We have retained many of the variable names that have been employed in previous models based on the DRAMA concept (see [19]). The organism physiology is described as a system of two pools (compartments) of carbon C: considered as a biomass-based model with units of mgC m^-3^, these are identified as the metabolite pool ^*M*^*C*, and the core biomass pool ^*C*^*C*. The total C-biomass of the organism is given by the sum of ^*C*^*C* and ^*M*^*C*. The acquired external resource firstly enters pool ^*M*^*C* (denoted by the thick pink horizontal arrow on the left of the diagram). The resource acquisition per capita (i.e., per the total C-biomass) rate is given by the term *A*_*max*_· ^*A*^*C*_*u*_, where *A*_*max*_ is the maximal rate of acquisition, and ^*A*^*C*_*u*_ (‘acquisition of carbon control’) is a function of ^*C*^*C* and ^*M*^*C*, which accounts for the decrease in resource acquisition due to feedback from the fullness of the metabolite pool ^*M*^*C* (see below the details about the parametrisation of ^*A*^*C*_*u*_). The maximal rate *A*_*max*_ is given by the product *A*_*max *_= *U*_*max*_· *A*_*m*_, where the parameter *U*_*max*_ is the maximal per capitate growth rate, and the coefficient *A*_*m*_ > 1 scales the acquisition rate above that required to support the maximal growth rate of the cell (*A*_*m*_ is time-dependent, since the resource acquisition depends on the light). The growth of the core biomass ^*C*^*C* occurs via synthesis at the expense of C flowing from ^*M*^*C*, which is described in the diagram by the thin horizontal pink arrow. The growth of ^*C*^*C* is modulated by the feedback from the relative fulness of pool ^*M*^*C*, described by the function ^*C*^*C*_*u*_ (‘consumption of carbon control’), which is mathematically a function of the relative fulness of pool ^*M*^*C* (see below). Therefore, the per capita growth rate of ^*C*^*C* is given by the product *U*_*max*_·^*C*^*C*_*u*_.

**Table 1:** Definitions of model variables, functions, parameters, units as well as their ranges and default values for the model. ‘DL’ denotes dimensionless variable/parameter.

Name	Meaning	Formulation, parameter range, unit, and if applicable default value
^*C*^ *C*	Core structural pool	mgC/m^3^
^*M*^ *C*	Metabolite pool	mgC/m^3^
*η*	Contribution of ^*M*^*C* to the total biomass	*η* =^ *M*^*C/*(^*C*^*C + *^*M*^*C*); DL
*A*_*m*_	Dependence of resource acquisition on light	$A_{m}=(1+B_{r}+C_{r})\cdot A_{0}$ during the light time; $A_{m}=0$ during the dark time; gC/gC/d
*R*	Relative size of the ^*M*^*C* pool to its maximum as set by *M*_*max*_	$R=min(1,max\;\left(0,\frac{\eta -M_{0}}{M_{\max}-M_{0}}\right))$; DL
^*C*^ *C*_*u*_	Feedback controlling growth of ^*C*^*C*, depending on the state of the ^*M*^*C* pool (‘consumption of carbon control’)	$CC_{u}=\left\{ \begin{array}{cc} (1+C_{uk}^{C_{uh}}fracR^{C_{uh}}R^{C_{uh}}+C_{uk}^{C_{uh}} & if\;R>0\\ -B_{r} & if\;R=0 \end{array}\right.\;$; DL
^*A*^ *C*_*u*_	Feedback controlling resource acquisition by the ^*M*^*C* pool (‘acquisition of carbon control’)	$AC_{u}=(1+A_{uk}^{Auh}frac(1-R{)}^{A_{uh}}(1-R{)}^{A_{uh}}+A_{uk}^{Auh}$, DL
Gr	Day-average growth rate	See expression (3); *d* ^-1^
${Gr}_{0}$	Normalised day-average growth rate	See expression (5); DL
*LD*	Proportion of the day that is illuminated	0≤LD≤1; DL
$A_{0}$	Adjustment in the resource acquisition rate required to support *U*_*max*_ when acquisition occurs only over the period *LD* relative to that when *LD* = 1	$1\leq A_{0}\leq 6;\;$DL
$A_{uh}$	Curve shape factor for ^*A*^*C*_*u*_	$A_{uh}$=2; DL
$A_{uk}$	Curve shape factor for ^*A*^*C*_*u*_	$A_{uk}$=0.05; DL
*B*_*r*_	Basal respiration rate expressed as a ratio to *U*_*max*_	*B*_*r*_ = 0.05; DL
$C_{uh}$	Curve shape factor for ^*C*^*C*_*u*_	$C_{uh}$=6; DL
$C_{uk}$	Curve shape factor for ^*C*^*C*_*u*_	$C_{uk}$=0.2; DL
*C*_*r*_	Anabolic respiration rate; this would actually vary depending on whether (for a phototroph) NH_4_^+^ or NO_3_^-^ was being assimilated	*C*_*r*_ = 0.2; gC/gC
*M*_*max*_	Maximum proportion of total carbon biomass occupied by ^*M*^*C*	$0.2\leq M_{\max}\leq 0.7$; DL
*M*_*o*_	Minimum proportion of organism biomass occupied by ^*M*^*C*	*M*_*o*_ = 0.05; DL
*U*_*max*_	Maximum per capita growth rate	$Ln(2)/2\leq U_{\max}\leq 4Ln(2);$ d ^-1^
*A*_*max*_	Maximum per capita rate of resource acquisition	*A*_*max*_* = U*_*max*_· ^*A*^*C*_*u*_ ·*A*_*m*_;; d ^-1^

We also take into account the respiration process due to catabolic (basal) and anabolic mechanisms, which is described in the model diagram by the vertical pink arrow. The basal respiration rate is described by the term *U*_*max*_· *B*_*r*_, where *B*_*r*_ is a positive parameter. The respiration due to anabolism is assumed to be proportional to the growth rate of the core biomass compartment, i.e., to be given by the product $C_{r}\cdot H{(}^{C}C_{u})\cdot^{C}C_{u}$, where the function *H* is mathematically the Heaviside step function (H(x)=1*,* x>0 and H(x)= 0 otherwise), the parameter $C_{r}$ describes the anabolic respiration rate. We also account for a return flow of carbon from ^*C*^*C* back to ^*M*^*C* in the case, where there is insufficient carbon in ^*M*^*C* to support even basal respiration; this is incorporated in the function *^C^C_u_* (see below). The model equations for the dynamics of ^*M*^*C* and ^*C*^*C* are given by


$$\begin{array}{c} \frac{d^{M}C}{dt}=U_{max}\left[{(}^{C}C{+}^{M}C)\cdot A_{m}\;\cdot^{A}C_{u}\;-B_{r}\cdot {(}^{C}C{+}^{M}C)-\;(C_{r}\cdot H{(}^{C}C_{u})+1)\cdot^{C}C_{u}\cdot^{C}C\right], \end{array}$$ (1)

$$\begin{array}{c} \frac{d^{C}C}{dt}={\;U}_{max}\cdot^{C}C_{u}\cdot CC \end{array}$$ (2)

In this study we use the following parameterisation of *A*_*m*_, ^*A*^*C*_*u*_, and ^*C*^*C*_*u*_. The time-dependent coefficient *A*_*m*_ (describing the scaling of resource acquisition) is parametrised as


$$A_{m}=\left\{ \begin{array}{cc} (1+B_{r}+C_{r}){\cdot A}_{0} & Dk\leq t<D(k+LD)\\ 0 & D(k+LD)\leq t<(k+1)D \end{array}\right.\;$$ (3)

which takes into account the light and dark periods. Here *D* is the length of a day (*D* = 1 d); *LD* is the proportion of the light time during the day (0≤ *LD* ≤1); *k* = 0,1, 2,... is the number of the day. The parameter $A_{0}$ provides adjustment in the resource acquisition rate sufficient to enable the maximum growth rate ($U_{\max}$) to be attained, when resource acquisition is allowed for only part of the day. For example, $A_{0}=2$ enables a maximum acquisition rate twice that required to support a maximum growth rate of $U_{\max}$ when acquisition is continuous. In this application, this parameter reflects the relative rate of resource acquisition during the light period but must also compensate for continued respiration during darkness.

The functions ^*A*^*C*_*u*_ and ^*C*^*C*_*u*_ relate to the state of fullness (satiation) of the ^*M*^*C* pool, denoted by *R* (see Fig 2B), between the minimum relative pool size, *M*_*0*_, and the maximum *M*_*max.*_


$$\begin{array}{c} R=\min\left(1,max\;\left(0,\frac{\eta -M_{0}}{M_{\max}-M_{0}}\right)\right),\; \end{array}$$ (4)

where *η*= ^*M*^*C*/(^*C*^*C*+ ^*M*^*C*).

The biological rationale of the parameter $M_{0}\;$ is that real organisms always contain a residual pool of metabolites in reflection of the continual recycling of materials with cell maintenance. The parameter $M_{\max}$ is the maximum proportion of the total biomass (^*C*^*C* +^* M*^*C*) occupied by the metabolite pool ^*M*^*C*. Using the above expression for *R*, we now introduce the following sigmoidal parameterisations for the functions ^*A*^*C*_*u*_ and ^*C*^*C*_*u*_*:*


$$CC_{u}=\left\{ \begin{array}{cc} (1+C_{uk}^{C_{uh}}fracR^{C_{uh}}R^{C_{uh}}+C_{uk}^{C_{uh}} & if\;R>0\\ -B_{r} & if\;R=0 \end{array}\right.\;,$$ (5)

$$\begin{array}{c} \;AC_{u}=\left(1+A_{uk}^{Auh}\right)\frac{(1-R{)}^{A_{uh}}}{(1-R{)}^{A_{uh}}+A_{uk}^{Auh}},\; \end{array}$$ (6)

where $C_{uh}$, $C_{uk}$, $A_{uh}$, and $A_{uk},\;$are model parameters. In particular, the value of ^*A*^*C*_*u*_ is maximal at low ratios of *R* and it drops to low values for a high fulness of the metabolic pool. The function ^*C*^*C*_*u*_ shows the opposite behaviour. Our argument for the choice of the sigmoid dependences is that operationally these are consistent with the allosteric nature of feedback-regulated processes in biological systems. Examples of graphs of ^*A*^*C*_*u*_ and ^*C*^*C*_*u*_, constructed as against the relative size of the metabolite pool *R* are shown in Fig 2B.

In this study, consistent with the types of empirical data seen in Fig 1, we are mostly interested in the average per capita growth rate *Gr* over the period (Δt) 1 day. To obtain the formula for *Gr*, we firstly sum up the time derivatives *d* ^*M*^*C/dt* and *d* ^*C*^*C/dt* given by (Eqs 1) and (2). Then we divide the obtained expression by the total biomass (^*C*^*C* + ^*M*^*C*) and perform integration over the interval Δt:


$$\begin{array}{c} Gr=\frac{U_{max}}{\Delta t}\int_{t}^{t+\Delta t}\left[A_{m}\;\cdot^{A}C_{u}(t)-B_{r}-C_{r}\cdot H{(}^{C}C_{u}(t))\cdot^{C}C_{u}(t)\frac{CC(t)}{CC(t){+}^{M}C(t)}\right]\;dt.\; \end{array}$$ (7)

Note that in (Eq 7), we integrate the difference between the resource acquisition rate and respiration. The total biomass in the model (^*C*^*C* +^* M*^*C*) should be understood as the total biomass of all organisms (e.g., mgC m^-3^) in the population, rather than that of an individual organism. As such, the above growth rate gives the population growth rate (i.e., C-specific growth rate, as C C^-1^ d^-1^).

It is easy to show that the expression for the growth rate depends on the ratio ^*M*^*C*/(^*C*^*C + *^*M*^*C*) or ^*C*^*C*/(^*C*^*C +*^* M*^*C*) rather than on ^*M*^*C* and ^*C*^*C* separately. Therefore, for the ratio *η* =^* M*^*C*/(^*C*^*C + *^*M*^*C*) we have the following dynamical equation:


dηdt=ddt(MCCC+MC)=dMC/dtCC+MC−CM(dCC/dt+dMC/dt)(CC+MC)2=dMC/dtCC+MC−η(dCC/dt+dMC/dt)CC+MC=(1−η)dMC/dtCC+MC−ηdCC/dtCC+MC (8)

We substitute the expressions for time derivatives $d^{M}C/dt$ and $d^{C}C/dt$ from model (Eqs 1) and (2) in the above expression to obtain


$$\begin{array}{c} \frac{d\eta }{dt}=U_{max}\cdot (1-\eta )\cdot \left[A_{m}\cdot AC_{u}-U_{max}\cdot B_{r}-\left(C_{r}\cdot H\left(CC_{u}\right)+1\right)\cdot (1-\eta )\cdot^{C}C_{u}-\eta^{C}{\cdot C}_{u}\right] \end{array}$$ (9)

Importantly, unlike *^M^C* and *^C^C*, the ratio *η* =^* M*^*C*/(^*C*^*C +*^* M*^*C*) is always bounded, and varies between 0 and 1. We should also note that both functions ^*A*^*C*_*u*_ and ^*C*^*C*_*u*_ (as well as *R*) depend on the ratio *η*. Therefore, expression of the average growth rate (Eq 3) would also depend on *η*. We can re-write the growth rate in a normalised form as


$${Gr}_{0}=\frac{Gr}{U_{\max}}=\frac{1}{\Delta t}\int_{t}^{t+\Delta t}\left[A_{m}\cdot AC_{u}(t)-B_{r}-C_{r}\cdot H{(}^{C}C_{u}(t))\cdot^{C}C_{u}(t)\cdot (1-\eta (t))\right]\;dt$$ (10)

In our study, we mostly considered equation (Eq 9) and integral (Eq 10) for *η* rather than the system (Eqs 1) and (2). Dynamic equation (Eq 9) and integral (Eq 10) were evaluated using standard numerical methods.

The model was implemented in both MATLAB and in Powersim Studio; the model using the latter is described in S1 File (see Tables A, B, C). For the considered parameter values (see Table 1 for detail), we found that the solution settles to a periodic attractor within a few days, which is due to strong external modulation of the resources availability, i.e., to the light-dark cycle. In particular, we did not find a strong influence of initial condition on the time to reach the periodic attractor. Therefore, we computed the value of ${Gr}_{0}$ after skipping the first few computational days (ca. 10 days) to ensure that the system reaches its asymptotic behaviour. We investigated the dependence of the normalised growth rate ${Gr}_{0}$ on the four key parameters $U_{\max}$, *LD*, $A_{0}$, $\begin{array}{c} M_{max} \end{array}$. We did not exhaustively investigate the consequences of using different values of the feedback control parameters ($C_{uh}$, $C_{uk}$, $A_{uh}$, and $A_{uk}$); as long as these describe response curves that show a wide level of overlap in saturation values across *R* (as seen in Fig 2B) the shape of these feedbacks has little consequence on general dynamics.

## Results

**Fig 3:**
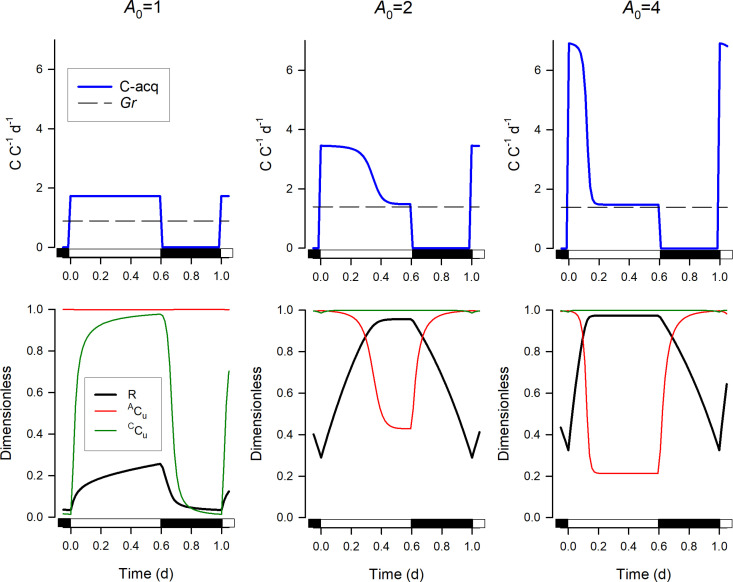
Examples of model outputs with the resource acquisition confined to 60% of the day (*LD* = 0.6; light in the first part of the indicated day). Upper panel: daily dynamics of the C-resource acquisition rate (C-acq) for different values of *A*_*0*_. The dashed line shows the day-average growth rate *Gr* given by (Eq 7). Bottom panel: the corresponding daily dynamics of ^*A*^*C*_*u*_, ^*C*^*C*_*u*_ and *R*. For all panels, the model parameters are $U_{\max}$= 1.386 *d*^-1^ (i.e., 2 doublings or divisions per day as Ln(4)) and *M*_*max*_^* *^= 0.6, the other parameters are provided in Table 1. See also Fig B from S2 File for examples where *A*_0_ is held constant, while *M*_max_ is varied.

Fig 3 shows the time course of typical model output over a day obtained using various values of *A*_*0*_. If *A*_*0*_ = 1 then the rate of acquisition can only support the maximum growth rate for *LD* = 1. For *A*_*0*_ = 2, the system exhibits growth at its maximal rate for *LD* > 0.5. Therefore, with *LD* = 0.6, as shown in Fig 3, the rate of acquisition is slowed part way through the light period by feedback from the fullness of the metabolite pool (due to the decrease of ^*A*^*C*_*u*_). With a higher value of *A*_*0*_ (*A*_*0*_ = 4), the metabolic pool fills very rapidly, such that feedback occurs earlier and the resource acquisition rate in the latter part of the light period is much lower, matching the rate of the flow of material from the compartment ^*C*^*M* to the compartment ^*C*^*C* after accounting for respiration, which is set by the maximal growth rate $U_{\max}$ (see Fig 2). In the upper panel of Fig 3 we also show the calculated day-average growth rates for the three different *A*_*0*_. In Figs B, C from S2 File, outputs are shown where *A*_0_ is held constant, while *M*_max_ is varied.

**Fig 4:**
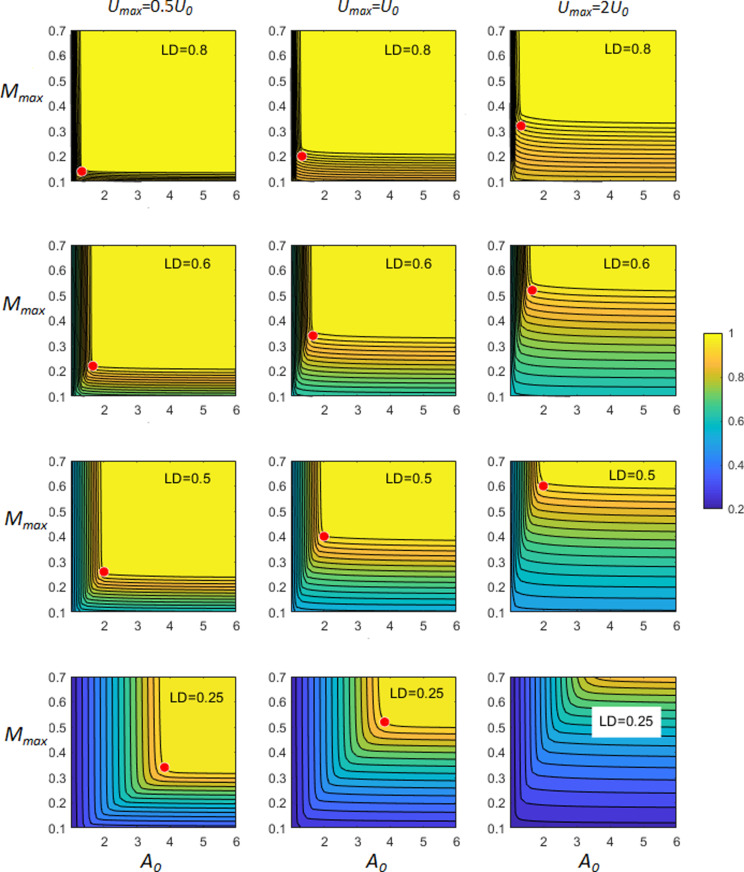
Example plots showing the normalised growth rate ${Gr}_{0}$ given by (Eq 10) achievable for given $A_{0}$, $M_{\max}\;$ values for an organism with resource acquisition limited to only that portion of the day indicated by *LD.* The maximal growth rates $U_{\max}$ are expressed as multiples of $U_{0},$ where $U_{0}=Ln(2)$ *d* ^-1^, corresponding to 1 division per day. The red dots indicate the optimum combination of values for $A_{0}$ and $\begin{array}{c} M_{max} \end{array}$, above which no further advantage is afforded to the organism under these scenarios.

**Fig 5:**
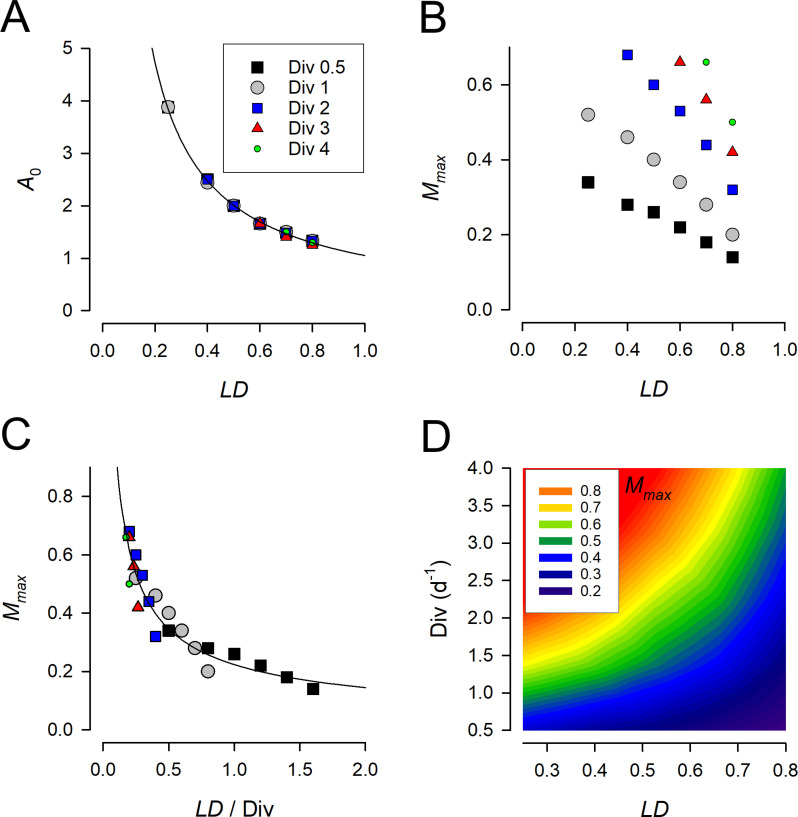
Summary of relationships between the parameters $A_{0}$ and $M_{max\;}$to deliver the optimal growth rate with resource acquisition only under a proportion of the day (*LD*). These values were derived from diagrams such as those shown in Fig 4; the data points in panels **(A)**, (**B**) and (**C**) have the same source, with those shown in (**C**) having being normalised to Div from panel **(A)**. The growth rate (*U*_*ma*x_) is described here as divisions per day, *Div*, as *U*_*ma*x_/Ln(2). The $M_{\max}$ scale in panel (**D**) is limited to 0.9, which is likely close to the plausible maximum contribution of metabolite-C to total-C, with the balance of C being allocated to core structural components. This suggests that much of the output space at *LD* < 0.5 is inaccessible to organisms with division rates of ca. > 2.5 d^-1^ (i.e., with ca. *U*_*max*_ > 1.7 d^-1^). The entire data series in panel **(A)**, indicated by the plotted line, is described by: *A*_0_ = 1.0511 *LD*^-0.935^, R^2^ = 0.9981. The entire data series in panel **(C)**, indicated by the plotted line, is described by: *M*_*max*_ = 0.2237 (*LD*/Div)^-0.629^, R^2^ = 0.9034.

We evaluated the day-average growth rate for different combination of model parameters $U_{\max}$, *LD*, $A_{0}$, $M_{\max}$. For each set of parameters, we ran simulations long enough to attain the periodic attractor. The results are presented in Fig 4 in the form of ${(A}_{0}$, $M_{\max}$) parametric diagrams constructed for different $U_{\max}$ and *LD*, where we plot the normalised growth rate ${Gr}_{0}$ defined by (Eq 10). This shows that for given *U*_*max*_ and *LD*, an increase in values of $A_{0}$ and $M_{\max}\;$above certain values (for fixed *U*_*max*_ and *LD*) provide no benefit in terms of enhancing the average growth rate: ${Gr}_{0}$ attains a plateau with values very close to 1, corresponding to $U_{\max}$. For each diagram, we also estimated the optimal values of $A_{0}$ and $M_{\max}$. Here we define the optimal values of $A_{0}$ and $M_{\max}$ as the minimal values for which the day-average growth rate attains 97.5% of $U_{\max}$. This modelling approach implicitly accounts for the underlying high costs of having larger values of these parameters for no substantial gain in the growth rate. In Fig 4 we denote such optimal values by red filled circles; they were found automatically by applying a standard optimisation procedure. These optimum values are re-plotted in Fig 5, to reveal the underlaying relationships.

We found that the optimal values of $A_{0}$ exhibit a non-linear increase with a decrease in the proportion of the light time *LD*, which can be mathematically approximated by a hyperbolic function, i.e., *A*_*0*_ ∝ *LD*^-1^ (see Fig 5A). On the contrary, the optimum value of $M_{\max}$at a given *LD* depends also on the maximal growth rate; the higher the value of *U*_*max*_, the larger the required optimal *M*_*m*_ (see Fig 5B). From Fig 5C it can be seen that the relationship defining $M_{\max}\;$comprises a series of linear relationships, one for each growth rate scenario. However, a curve fitted to these data describes 90% of the relationship between $M_{\max}$ and *LD*/*Div* (here *Div* is the number of divisions per day). Finally, the corresponding contour plot relating $M_{\max}$to *Div* and *LD* is presented in Fig 5D. It is noteworthy that much of the space at low *LD* is inaccessible to fast growing configurations, since it requires an implausibly high value of $M_{\max}$.

Some example outputs of the model using the optimised parameters from relationships in Fig 5 are shown in Fig C (from S2 File) for comparison with Fig 3 and Fig B. These show how the feedback processes act to maintain similar internal conditions, as reflected by the values of *R*, ^*A*^*C*_*u*_ and ^*C*^*C*_*u*_.

## Discussion

It is more important, and more efficient, for an organism’s health to maintain a steady metabolic status (homeostasis) than to switch processes on and off. Accordingly, when confronted by conditions in which resources are available discontinuously, for periods of time, it is preferable to acquire those resources as and when possible, and then convert them into organismal growth over time at a steadier rate. The duration of the cell-cycle (through G1, S, G2 and M stages) will also constrain organismal growth operations. In consequence, the growth of organisms in diel cycles of different duration (e.g., for the microalgae shown in Fig 1, [20,21] may not be well described as a simple relationship between their day-average growth rate and the period of resource acquisition. To appropriately model such a mechanism to function for the acquisition of a dominant component of the organism’s biochemistry, notably for carbon, requires both a capability to acquire the resource very rapidly when the opportunity arises, and also to retain the capacity to accumulate the immediate products of acquisition.

In this study, we explore this interaction using a simple 2-pool model (Fig 2A) through which recently acquired nutrients are held and exploited in a metabolite pool in support of the growth of the second pool over a longer time at a steadier rate. While the use of intermediate pools to accumulate nutrient reserves has been considered in models before [18,24–26], our study is unique as it considers both feedbacks controlling resource acquisition and its use for growth, and also considers the acquisition of the most important single resource, namely C. Both the relative excess rate of resource acquisition over the day-average need (set by the parameter *A*_*0*_ in the model), and the maximum size of the metabolite pool into which the resource is initially deposited (set by the parameter *M*_*max*_) affect the shape of the relationship between the periodicity of resource availability and the relative growth rate attained. The synthesis of ^*C*^*C* depends on materials flowing from ^*M*^*C*; that rate is capped by *U*_*max*_. Here, we assumed that the flow of carbon into ^*C*^*C*, interrupted by *LD*, was the only limiting factor. In reality, there are factors other than that carbon flow that may restrict the use of metabolites, notably the acquisition of other nutrients. In a multi-currency model (e.g., C,N,P) the synthesis of ^*C*^*C* is thus potentially constrained by a multitude of interactions, but there is no negative feedback from the relative size of ^*C*^*C* (i.e., ^*C*^*C*/(^*C*^*C* + ^*M*^*C*)) as such. For ^*C*^*C* to ‘over-fill’, by definition ^*M*^*C* must be relatively small and thus limiting. The relative size of the metabolite pool is a key feature of the model; in multi-nutrient DRAMA descriptions this size is used to modulate (regulate) various features [19], but the importance of *M*_*max*_ is shown by our results to itself be of importance in controlling growth dynamics in situations where the C-resource (here, as light for photosynthesis) is provided discontinuously.

Considered here when applied to the limiting resource to be C derived from an optimal rate of photosynthesis during the light period, we located interactions between *A*_*0*_ and *M*_*max*_ with *U*_*max*_ and *LD* (Figs 4, 5). The model predicts that the higher the potential growth rate, *U*_*ma*x_, the higher both the optimal acquisition rate and also *M*_*max*_ must also be for a given *LD*. These conditions must be met to enable a high value of the ratio *R* (the relative size of the ^*C*^*M* pool) to be attained over a given proportion of the light time *LD*. The cost of achieving such metabolic flexibility depends then upon the fluctuation of *LD* in the environment in which the organism evolves, and the material and operation costs of *A*_*0*_ and *M*_*max*_. For a phototroph, the total costs of the photosynthetic machinery is significant [27]; in the model this equates to enabling *A*_*max*_ = *U*_*ma*x_ x *A*_*0*_, as the resource acquisition rate. From Fig 5A it can be seen that at low *LD* this rate becomes very high. Although one could argue that the proceeds of carbon fixation themselves pay for such a cost, there are additional overheads that must be met for a real organism. These are most obviously nitrogen (for proteins) and also, of especial concern in certain areas of the ocean, for the Fe required in co-factors of the light-reactions resulting in a strong relationship between the Fe:C quota and growth irradiance [28,29].

The main emphasis on the modelling of photoacclimation in microalgae has been on changes in Chl:C in response to nutritional status and irradiance [8,30]. Acclimations to variation in *LD* are not considered. This likely reflects a bias in laboratory studies in maintaining a constant LD during culture work while studying the effects of just changing irradiance or interactions with nutrient status [31]. Our work suggests that the value of *M*_*max*_ might be of equal, if not greater, concern than C-fixation in terms of resource allocation for fast growing microalgae in low *LD* conditions. From the contour plot in Fig 5D it is apparent that growth rates above a division per day (*U*_*max*_ = 0.693 *d*^*-1*^) at *LD* = 0.5 already require ca. 40% of the cellular C to be accumulated in the form of readily metabolizable materials. This growth rate is common, with the division cycle confined to the dark period of growth (i.e., night time; [2]). For organisms capable of growing with 2 divisions per day, such as diatoms and coccolithophorids, such an allocation of space to ^*C*^*M* would only permit such a growth rate with *LD* > ca.0.7 (e.g., [20,21]; Fig 1). This structural demand thus places an important control on the spatial and temporal emergent maximum growth rate and seasonality bounds for phototrophic plankton. If one argues that larger diatoms, which are relatively more vacuolated [32–34], have more space for elevated *M*_*max*_, then one may expect such organisms to be at an advantage in low *LD* conditions. Mixoplankton, which combine phototrophy and phagotrophy [35], may be able to mitigate against such challenges through prey consumption.

From the equation describing the interactions between division rate, *LD* and *M*_*max*_ (legend Fig 5), it is seen that at extremely low *LD* it becomes impossible for a phototroph to accumulate a significant amount of C in the light period (the rate of photosynthesis, as defined by *A*_*0*_ × *U*_*max*_*,* cannot deliver to the need – Fig 5A) and/or there is no space to accumulate the intermediates (*M*_*max*_ is limiting). At the other extreme, in continuous light with *LD* = 1, there is in modelling terms no justification for describing a metabolite pool and *A*_*0*_ = 1. In reality, of course, there would always be a metabolite pool. For organisms with a very low *U*_*max*_, the required value of *M*_*max*_ may be small, even at low *LD* (see Fig 5D).

With global climate change, water temperatures are increasing [36]. This will affect the expressed *U*_*max*_ for plankton [37] and, unless the species alter their seasonality or evolve their expressed *U*_*max*_ downwards [38], they will become relatively more stressed if their space allocations for *M*_*max*_ required for them to attain high growth rates become limiting. That may be even more likely as microalgae grown at elevated temperatures tend to be smaller [39] and may have less scope for expressing a high *M*_*max*_. The situation may be more problematic again, because in our study we assume that resource availability itself is not limiting in any way during the light period. In practice, for example, the photon flux density varies greatly during the day; it would be logical for an organism to be able to express significantly higher than the steady-state optimum acquisition rate to make the most of what light is available when it is available. The dynamics of such an excess rate of acquisition can be seen in Fig 3 for *A*_*0*_ = 4; here the high acquisition rates during morning photosynthesis results in the rapid filling of the metabolite pool which then depressed the acquisition during the afternoon. This behaviour of the model is consistent with observations that afternoon rates of photosynthesis are depressed relative to those in the morning but that this relationship is most obvious when the initial photosynthetic rate is high [40].

In addition to the subcellular space challenges of expressing a large *M*_*max*_ for faster growing organisms at a given *LD* (except when *LD* = 1, when *M*_*max*_ can in theory equate to *M*_*0*_), there is the liability associated with the increased leakage of metabolites from such a large metabolite pool into the water which may then support the growth of competitors and also attract predators [41]. Our results suggest that growing slowly (i.e., have a lower *U*_*max*_) is of benefit for an organism exploiting a range of conditions affecting the temporal availability of resources. This provides additional evidence to support the growth rate evolution model from [38], which has as its core tenant that an ability to express high growth rates comes at a cost, and a failure to meet physiological demand with resource supply results in stress and thence death.

From a modelling point of view, the addition of another state variable (i.e., by dividing the carbon biomass description into state variables ^*M*^*C* and ^C^*C,* or equivalently, using the ratio *η* = ^*M*^*C*/(^C^*C* + ^*M*^*C*)) with its computational overhead and parameterisation challenges, could be seen as undesirable by those running complex models. For such models (at the extreme, Earth Systems Models) every additional state variable added per organism described may be seen as computationally costly. The parameterisation challenge itself is minor; Fig 5 provides a guidance. It should be noted, however, that in reality the availability of light is most often sub-optimal, either too low or too high and can vary greatly over the daylight hours, prompting various responses [42]. As there is no way for an organism to foresee how the day’s illumination regime will develop, one may expect a microalga to express (in the terms used in our model) larger values for *A*_0_ than indicated by Fig 5A. Exploitation of such extreme acquisition rates underscore the value of possessing a suitably high *M*_*max*_, a trait that thus appears as an important selective criterion that warrants expression in models of these organisms.

Inclusion of the state variable ^*M*^*C* in essence provides a description of a C-quota which is analogous to the other nutrient quotas (i.e., N:C, P:C, Fe:C) commonly used in plankton models [11]. In common with the use of other quotas, the use of the *η* = ^*M*^*C*/(^C^*C* + ^*M*^*C*) quota describes growth relating to internal rather than directly to external resource availability. Addition of this C-quota also enables an ability to modulate resource acquisitions from different routes, which is particularly relevant when considering mixotrophic activity [35] that combines phototrophy and heterotrophy (osmotrophy of dissolved organics and/or phagotrophy of particulate organics including prey). We argue that the additional computational cost of including this state variable (a cost that could be considered as significant in large scale 3D models) is justified by the significant advance in describing plankton physiology that is thus enabled.

What of other organisms, how may these be affected by the constraints shown by our model? Many plankton species perform diel vertical migration, either downwards for phototrophs to obtain nutrients at night [43,44], or upwards for zooplankton to feed [45,46]. These migrating plankton species are, in the main, organisms with biomass doublings significantly less than 1 per day (e.g., copepods have C-specific growth rates of ca. 0.2 d^-1^; [47]). Even if feeding was limited to a rather small fraction of a day, modelling of the relatively low rates of growth for a copepod would (from Fig 5D) only warrant *M*_*max*_ < 0.2. However, similarly to the situation for the availability of light for phototrophy in the real world, discussed above, predation for these organisms is unlikely to proceed at a constant rate, complicated further by prey selection [48]. In reality, then, an ability to rapidly consume a temporary super-abundance of a suitable resource (requiring in our terminology a high *A*_0_), and an appropriately high *M*_*max*_ would be advantageous. Modelling of such behaviour may also benefit from such considerations.

To conclude, for the computational cost of including an extra C-state variable, such that C-biomass is split between a metabolite pool and a core structural pool, it becomes possible to provide a model that can better describe the short-term dynamics of resource acquisition at rates far exceeding the day-average needs. This is a necessary advance for exploring the competitive advantages between species growing under different environmental conditions; this includes models describing the primary production undertaken by the planktonic phototrophs that support food chains and biogeochemical activities across over 2/3^rds^ of Earth.

## Supporting information

S1 File Description of the model simulation using Powersim Studio software (Fig A, Tables A,B,C). (DOCX)

S2 File Supplementary figures (Fig B, Fig C, Fig D), describing model outputs for daily dynamics of the C-resource acquisition rate and daily dynamics of *R*, ^*A*^*C*_*u*_ and ^*C*^*C*_*u*_. (DOCX)
