# Supplementary material for: Resource acquisition in diel cycles and the cost of growing quickly
Source: PLoS Comput Biol. 2025 Jun 6;21(6):e1013132. doi: 10.1371/journal.pcbi.1013132 (PMC12803028; doi:10.1371/journal.pcbi.1013132)
Supplement: S1 File — (DOCX) [file pcbi.1013132.s001.docx]

# SUPPORTING INFORMATION: Resource acquisition in diel cycles and the cost of growing quickly.

Kevin J. Flynn^1^, Andrey Yu. Morozov^2,3*^

^1^Plymouth Marine Laboratory, Plymouth, UK

^2^School of Computing and Mathematical Sciences, University of Leicester, Leicester, UK

^3^Institute of Ecology and Evolution, Russian Academy of Sciences, Moscow, Russia

*Corresponding author: am379@leicester.ac.uk

## S1. Model simulation using Powersim Studio.

The following describes the Forrester diagram and variable descriptions required to build the model in Powersim Studio 10. The diagram can be readily compared with the conceptual schematic in **Fig 2**.

In the diagram, state variables are rectangles, constants are diamonds, and intermediate terms (‘auxiliaries’) as circles with their required inputs indicated by arrows. Flows are pipelines. Arrows in and out of symbols in the Forrester diagram indicate import and export of data to Excel. The model was operated under a Euler integration routine with the step size of 0.0078125 d. The dilution term enables the model to be run in a chemostat mode.

**Fig A**. Flowchart of the Forrester diagram of the model implemented using the Powersim Studio 10 software.

**State variables**

The definitions give example for initial values, followed by flows in (‘+’) and out (‘-‘); see also the pipelines in the Forrester diagram (see Table A).

Table A. State variables and the growth rate, as well as the initial values used in modelling based on Powersim Studio 10 software. Both notations are provided: for the Powersim Studio 10 software and for the mathematical equations in the main text

| **Name**  **(Powersim Studio)** | **Name**  **(main text equations)** | **Definition** | **Unit and Documentation** |
| --- | --- | --- | --- |
| CC | *^C^C* | 1e-6; {+syn - CC_dil} | mgC/m^3^ core structural pool |
| MC | *^M^C* | CC*M0; {+A - syn - resp - CM_dil} | mgC/m^3^ metabolite pool |

Table Bbelow provides the description of the model parameters used in Powersim Studio 10code.

Table B. Definition of model ‘constant’ parameters and their units used in Powersim Studio 10 to run the model. For convenience, we also provide the corresponding notation used in the model equations in the main text. The symbol ‘**’ means that this parameter is only used in Powersim Studio 10 code.

| **Name**  **(Powersim Studio)** | **Name**  **(main text equations)** | **Default value** | **Unit and Documentation** |
| --- | --- | --- | --- |
| A0 | $A_{0}$ | 4 | DL ratio of resources acquisition rate required to support a gross input vs Umax |
| AuH | $A_{uh}$ | 2 | DL curve shape factor for ACu |
| AuK | $A_{uk}$ | 0.05 | DL curve shape factor for ACu |
| Br | *B_r_* | 0.05 | DL basal respiration rate expressed as a ratio to Umax |
| Cr | *C_r_* | 0.2 | gC/gC anabolic respiration rate; this would actually vary depending on whether (for a phototroph) NH4 or NO3 was being assimilated |
| CuH | $C_{uh}$ | 6 | DL curve shape factor for CCu |
| CuK | $C_{uk}$ | 0.2 | DL curve shape factor for CCU |
| dil | ** | 0.05 | d^-1^ dilution rate (used only in Powersim Studio for technical purposes) |
| M0 | *M_o_* | 0.05 | DL minimum proportion of organism biomass occupied by MC; in a real organism there is always a small pool used to support protein turnover etc. Pragmatically, this also prevents MC from running below 0 at high integration step sizes) |
| Mmax | *M_max_* | 0.6 | DL maximum proportion of total C biomass occupied by MC |
| Umax | *U_max_* | 1 | gC/gC/d maximum growth rate (technically valid at the current temperature only) |

Table Cbelow provides description of the auxiliaries i.e., the terms of in the model equations, used in the Powersim Studio 10 software.

TableC. Auxiliaries needed to run the model in Powersim Studio 10. The symbol ‘**’ means that this auxiliary quantity is only used in Powersim Studio 10 code.

| **Name**  **(Powersim Studio)** | **Name**  **(main text equations)** | **Definition** | **Unit and Documentation** |
| --- | --- | --- | --- |
| A | (*^M^C+ ^C^C*)*A_max_* | TC*Am*ACu* Umax | mgC/m^3^/d rate of C acquisition, filing MC |
| ACu | *^A^C_u_* | (1+AuK^AuH)*(1-R)^AuH/((1-R)^AuH+AuK^AuH) | DL sigmoidal feedback curve controlling resource acquisition from the state of the MC pool |
| Am | *A_m_* | IF(FRAC(TIME)<LDop, *(1+Br+Cr)*A0) | DL Dependence of resource acquisition on light; when A0=1, this enables growth at 1 with LD=1 |
| AsCu | ** | A/TC | gC/gC/d C-specific resource acquisition rate |
| CC_dil | ** | CC*dil | mgC/m^3^/d dilution of CC |
| CCu | *^C^C_u_* | (1+CuK^CuH)*R^CuH/(R^CuH+CuK^CuH) | DL sigmoidal feedback controlling growth of CC, exploiting metabolites in MC, depending on the state of the MC pool |
| Cu | ** | AsCu-resCu | gC/gC/d C-specific growth rate |
| Cu_I | ** | Cu | gC/gC/d current growth rate |
| Cu_O | ** | DELAYPPL(Cu_I,1,0) | gC/gC/d growth rate 1 day ago |
| Gr | $Gr$ | Cu_I - Cu_0 | gC/gC/d day-average growth rate |
| Gr0 | ${Gr}_{0}$ | Gr/Umax | DL day-average relative growth rate |
| LD | *LD* | 1 | DL L:D ratio as a proportion of the day that is illuminated |
| LDop | ** | LD | DL L:D ratio as a proportion of the day that is illuminated (this variable is declared to overcome an I/O feature of Studio) |
| R | *R* | MIN(1,MAX(0,(MC/TC-M0)/(Mmax-M0))) | DL relative size of the MC pool to its maximum as set by Mmax |
| resCu | ** | resp/TC | gC/gC/d C-specific respiration rate |
| resp | ** | Umax*Br*TC+IF(syn>0,Cr*syn) | mgC/m^3^/d respiration rate removing C from MC |
| syn | ** | CC*(Umax*CCu-IF(CCu=0,Umax*Br)) | mgC/m^3^/d rate of synthesis of CC exploiting metabolites from MC. This includes a return of C from CC to MC if there is insufficient C entering MC to meet respiratory demands |
| TC | ** | CC+MC | mgC/m^3^ total organism biomass |
