# Supplementary material for: Resource acquisition in diel cycles and the cost of growing quickly
Source: PLoS Comput Biol. 2025 Jun 6;21(6):e1013132. doi: 10.1371/journal.pcbi.1013132 (PMC12803028; doi:10.1371/journal.pcbi.1013132)
Supplement: S2 File — (DOCX) [file pcbi.1013132.s002.docx]

## S2. Supplementary Figures with Simulation Results


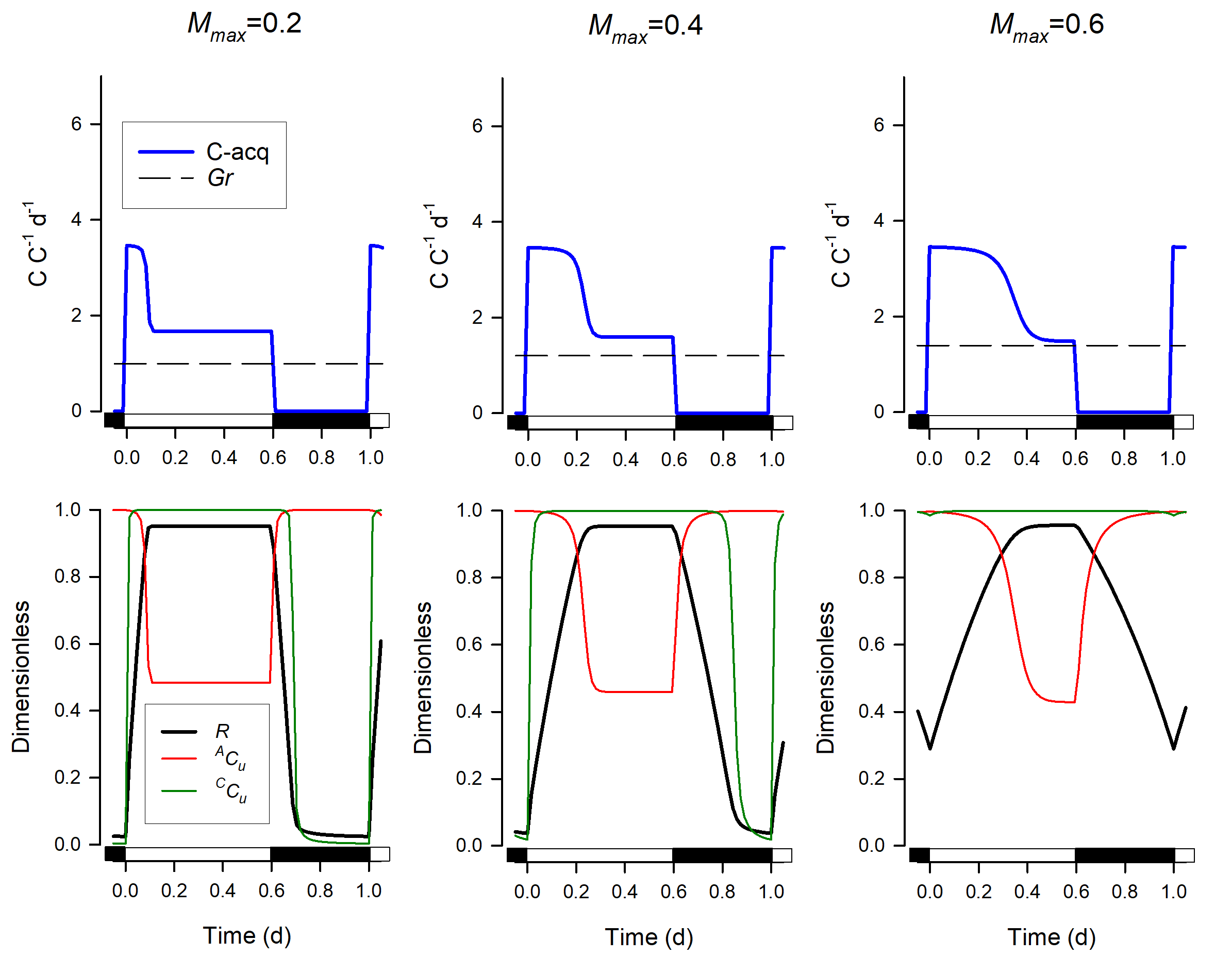


**Fig B.** For comparison with **Fig 3** from the main text, examples of model output with the resource acquisition confined to 60% of the day (*LD* = 0.6; light in the first part of the indicated day) but with *A*_0_ held constant, while *M*_max_ is varied. Upper panel. Daily dynamics of the C-resource acquisition rate (C-acq) for different values of *M_max_*. The dashed line shows the day-average growth rate *Gr* given by (Eq 7). Bottom panel. The corresponding daily dynamics of *^A^C_u_*, *^C^C_u_* and *R*. For all panels, the model parameters are $U_{max}$= 1.386 *d*^-1^ (i.e., 2 doublings or divisions per day) and *A*_0_ = 2, the other parameters are provided in **Table 1**.


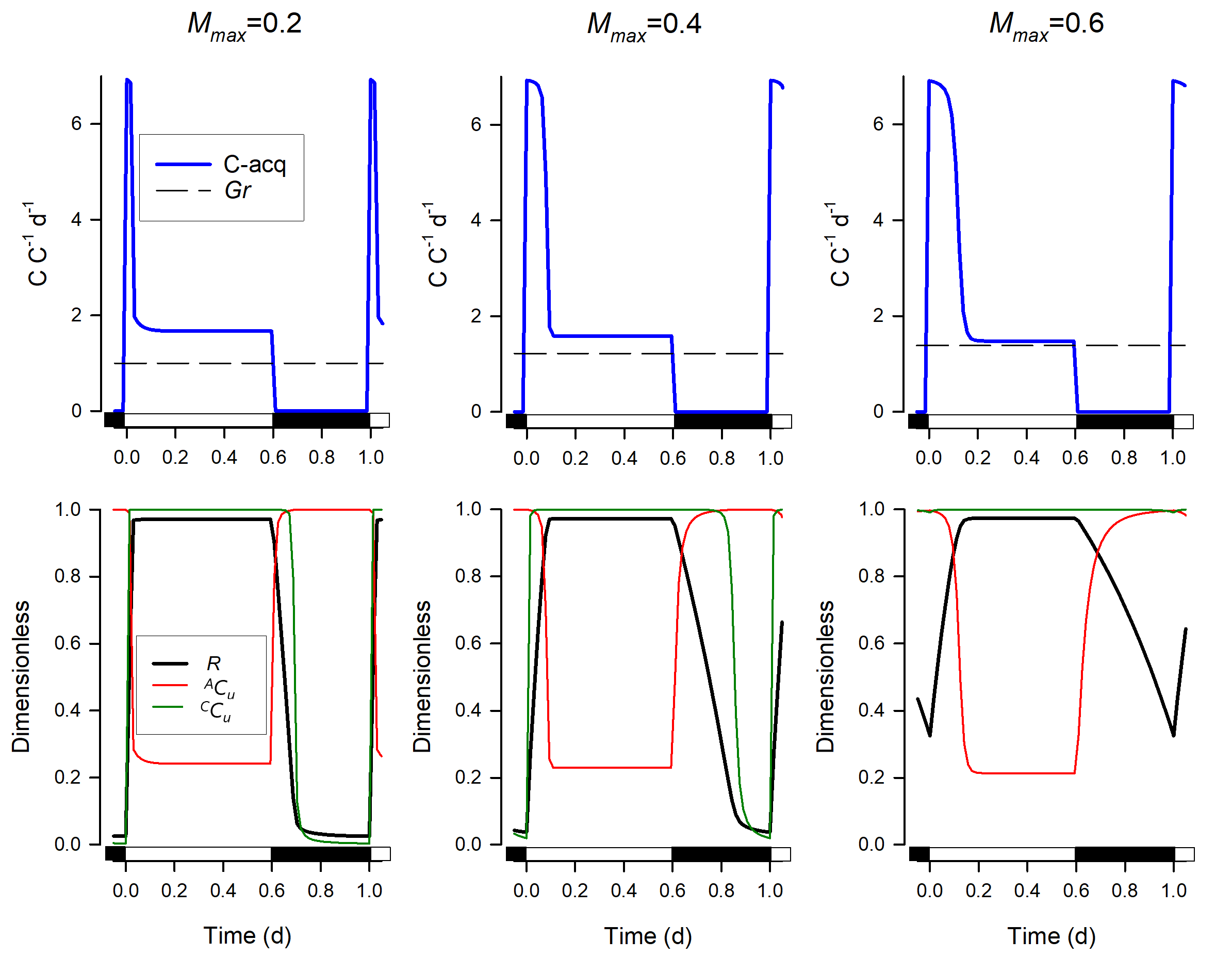


**Fig C.** For comparison with **Fig 3** from the main text, examples of model output with the resource acquisition confined to 60% of the day (*LD* = 0.6; light in the first part of the indicated day) but with *A*_0_ held constant, while *M*_max_ is varied. Upper panel. Daily dynamics of the C-resource acquisition rate (C-acq) for different values of *M_max_*. The dashed line shows the day-average growth rate *Gr* given by (Eq 7). Bottom panel. The corresponding daily dynamics of *^A^C_u_*, *^C^C_u_* and *R*. For all panels, the model parameters are $U_{max}$= 1.386 *d*^-1^ (i.e., 2 doublings or divisions per day) and *A*_0_ = 4, the other parameters are provided in **Table 1**.

**
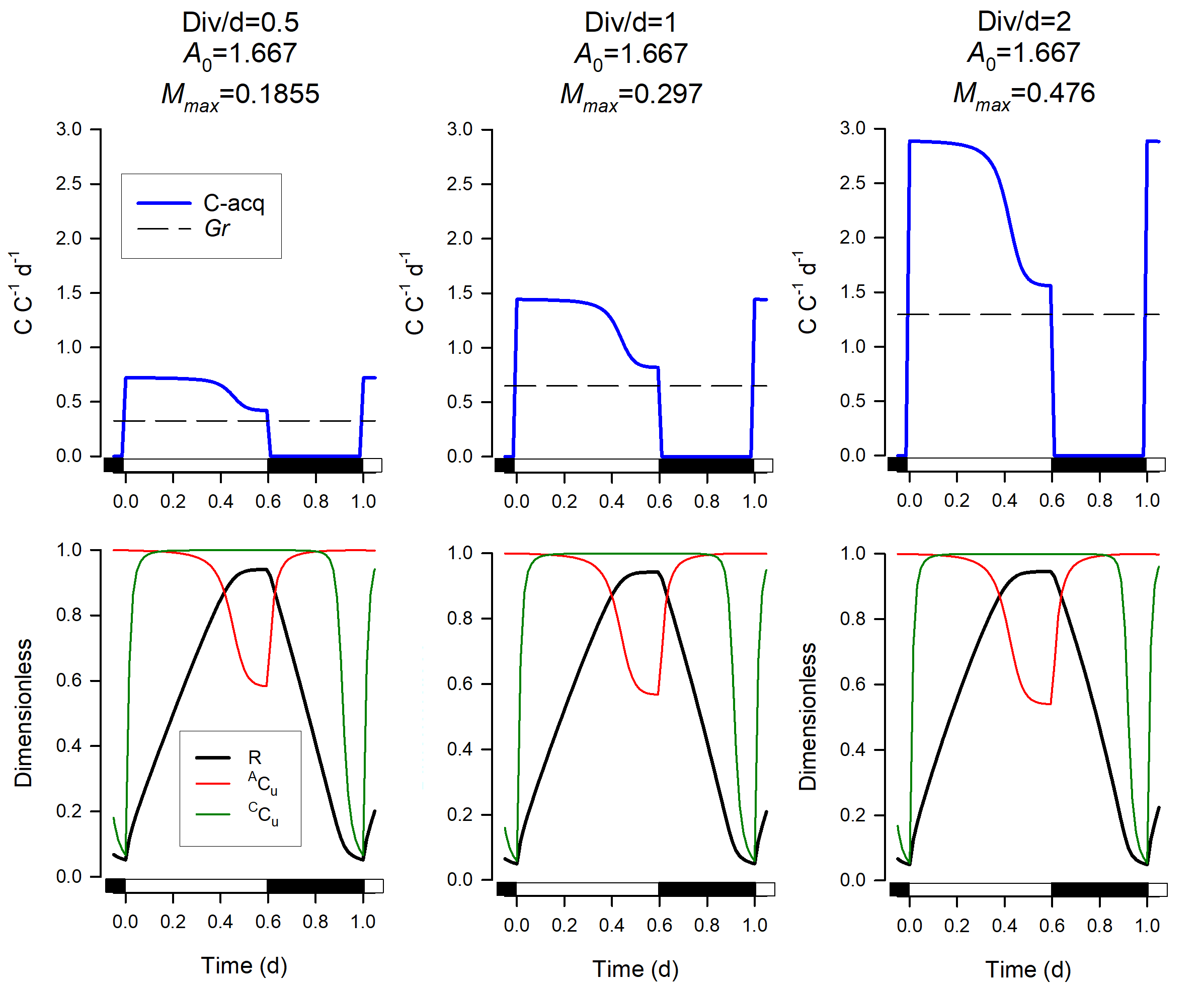
**

**Fig D.** For comparison with **Fig 3** (main text) & **Figs**. **B**,**C** examples of model output using values of *A*_0_ and *M_max_* derived from the relationships shown in **Fig 5** from the main text (as shown in the headers for each column). Note the similarities between the lower panels. ‘Div/d’ – divisions per day; a value of 1 equates to a day-average C-specific growth rate of 0.693 *d*^-1^.
